# BabA-mediated adherence of pediatric ulcerogenic *H. pylori* strains to gastric mucins at neutral and acidic pH

**DOI:** 10.1080/21505594.2018.1532243

**Published:** 2018-11-05

**Authors:** Macarena P. Quintana-Hayashi, Raquel Rocha, Médea Padra, Anders Thorell, Chunsheng Jin, Niclas G. Karlsson, Mónica Roxo-Rosa, Mónica Oleastro, Sara K. Lindén

**Affiliations:** a Department of Biomedical Chemistry and Cell Biology, Institute of Biomedicine, Sahlgrenska Academy, University of Gothenburg, Gothenburg, Sweden; b Department of Infectious Diseases, National Institute of Health Dr. Ricardo Jorge, Lisbon, Portugal; c Department for Clinical Science and Department of Surgery, Ersta Hospital, Karolinska Institutet, Stockholm, Sweden; d Centro de Estudo de Doenças Crónicas, Nova Medical School/Faculdade de Ciências Médicas, Universidade Nova de Lisboa, Lisbon, Portugal

**Keywords:** Mucin, *Helicobacter pylori*, peptic ulcer, adhesin, babA, children, binding, virulence, pH, glycosylation

## Abstract

*Helicobacter pylori* infection can result in non-ulcer dyspepsia (NUD), peptic ulcer disease (PUD), adenocarcinoma, and gastric lymphoma. *H. pylori* reside within the gastric mucus layer, mainly composed of mucins carrying an array of glycan structures that can serve as bacterial adhesion epitopes. The aim of the present study was to characterize the binding ability, adhesion modes, and growth of *H. pylori* strains from pediatric patients with NUD and PUD to gastric mucins. Our results showed an increased adhesion capacity of pediatric PUD *H. pylori* strains to human and rhesus monkey gastric mucins compared to the NUD strains both at neutral and acidic pH, regardless if the mucins were positive for Lewis b (Le^b^), Sialyl-Lewis x (SLe^x^) or LacdiNAc. In addition to *babA* positive strains being more common among PUD associated strains, *H. pylori babA* positive strains bound more avidly to gastric mucins than NUD *babA* positive strains at acidic pH. Binding to Le^b^ was higher among *babA* positive PUD *H. pylori* strains compared to NUD strains at neutral, but not acidic, pH. PUD derived *babA*-knockout mutants had attenuated binding to mucins and Le^b^ at acidic and neutral pH, and to SLe^x^ and DNA at acidic pH. The results highlight the role of BabA-mediated adherence of pediatric ulcerogenic *H. pylori* strains, and points to a role for BabA in adhesion to charged structures at acidic pH, separate from its specific blood group binding activity.

## Introduction

In half of the human population, the stomach is colonized by the pathogen *Helicobacter pylori* (*H. pylori*). *H. pylori* infection generally occurs at an early age resulting in a long-term inflammation in the gastric mucosa. Although infection can be asymptomatic, infected patients may develop non-ulcer dyspepsia (NUD), peptic ulcer disease (PUD), adenocarcinoma, and mucosa-associated lymphoid tissue (MALT) lymphoma[1-3]. PUD includes both duodenal ulcer (DU), as a result of antral infection and acid hypersecretion, and gastric ulcer (GU) caused by gastric atrophy and acid hyposecretion [1].In pediatric patients PUD is rare and occurs shortly after infection, therefore its pathogenesis is most likely less dependent on environmental factors and more importance is given to strain virulence determinants. Genotyping of *H. pylori* isolates has revealed certain bacterial genes to be associated with clinical outcome [4,5]. *HomB* expression is associated with PUD in both children and adults, whereas *homA* is associated with non-ulcer dyspepsia (NUD) [4,6]. Furthermore, *homB* has been suggested to play a role in the inflammatory response and *H. pylori* adherence to gastric epithelial cells [4].

The mucus layer that covers mucosal surfaces is the first barrier that *H. pylori* encounter, and is the niche in which most *H. pylori* are localized. The mucus layer in the healthy stomach consists mainly of the gel-forming mucins MUC5AC, secreted from the superficial mucosa, and MUC6 secreted from the gland mucosa [7]. In addition to the gel-forming mucins, the mucus layer also contains the shed extracellular domain of the cell-surface mucin MUC1, DNA from sloughed off cells, and a range of antimicrobial molecules [8].

*H. pylori* uses a range of binding modes to adhere to the highly glycosylated mucins; via the blood group antigen binding adhesin (BabA) that binds to Lewis b (Le^b^) and related structures, via the sialic acid binding adhesin (SabA) that binds to sialyl-Lewis x (SLe^x^) and sialyl-Lewis a (SLe^a^), and via a charge/low pH dependent mechanism [9-11]. Furthermore, *H. pylori* has been suggested to bind to the GalNAcβ1-4GlcNAc motif (lacdiNAc) via LabA [12], and both *H. pylori* and its close relative *H. suis* bind to Lacto-N-tetraose (LNT,Galβ3GlcNAcβ3Galβ4Glc), present on gastric glycolipids and on mucins [13].The adhesion targets and the glycan environment that *H. pylori* is exposed to differ between individuals and become more sialylated in response to *H. pylori* infection and disease development [11,14-19]. The presence of the *H. pylori* adhesins BabA and SabA hasbeen associated with a more severe disease outcome. However, in the *H. pylori* rhesus macaque infection model, BabA expression has been shown to decrease within weeks of infection due to selective pressure, although BabA was required for establishment of infection [20].

The glycan structures to which *H. pylori* binds can be present both on glycolipids and mucins, with the former most likely conferring a more intimate and disease promoting adhesion and the latter providing a decoy and defense system [9,21,22]. Indeed, *H. pylori*-infected rhesus monkeys and human children secreting mucins with lower *H. pylori* binding ability develop higher *H. pylori* density infections and gastritis [19,23]; suggesting that binding of *H. pylori* to secreted mucins protects the gastric epithelium. Gastric mucin turnover is impaired during *H. pylori* infection, creating a more stable environment for long term colonization [24].

To our knowledge, the interactions of *H. pylori* strains isolated from children with gastric mucins remain unknown. Therefore, we characterized the binding ability and adhesion modes of *H. pylori* strains from pediatric patients with NUD and PUD to human and monkey gastric mucins at acidic and neutral pH. The mucins were selected based on their differential display of glycan epitopes relevant for interactions with *H. pylori*. Our results demonstrated increased adherence of PUD *H. pylori* strains to gastric mucins compared to the NUD strains at both neutral and acidic pH. We therefore investigated the contribution of previously described *H. pylori* binding modes (BabA, SabA, LabA, HomB and the charge dependent mechanism) to this difference in binding. The results highlight the role of BabA-mediated adherence of pediatric ulcerogenic *H. pylori* strains and suggests a role for BabA in adhesion to charged structures at acidic pH, separate from its specific blood group binding activity that has a neutral pH optimum.

## Results

### Genotypic characterization of NUD and PUD H. pylori virulence factors

In children peptic ulcer disease occurs shortly after infection, hence *H. pylori* virulence determinants have been suggested to be important in disease development [25]. The genetic characterization of *H. pylori* clinical isolates has shown an association between certain bacterial genes (*e.g. homB* and *jhp562*) and the development of PUD in children [4,6,25]. Therefore, the *H. pylori* strains derived from NUD (n = 9) and PUD (n = 11) pediatric patients were genotyped for the presence of putative virulence genes *babA, babB, babC, cagA, vacAs, hopQ* allele, *oipA, sabA, hopZ*, and *homB* byWGS (Table 1).The majority of the PUD strains had a virulence profile characterized by the presence of *babA* (8/11), *babB* (10/11), *babC* (8/11),*cagA* (11/11), *homB* (10/11), *vacA*s*1* (10/11), *hopQI* (8/11), *labA* (8/11), and carried the *oipA* (10/11) and *hopZ* (8/11) functional “on” status, while *sabA* functional “on” status was not a dominant characteristic (5/11).On the contrary, most NUD *H. pylori* strains were negative for *babA* (7/9), *babC* (6/9), *cagA* (9/9), *homB* (7/9), had *oipA* functional status “off” (9/9), presence of *vacA*s *2*(9/9), *labA* (9/9), and *sabA* functional status “on” (6/9).Overall, the results show that NUD and PUD *H. pylori* pediatric strains display distinct virulence profiles, with PUD strains expressing several virulence-associated genes such as those encoding the vacuolating cytotoxin VacA, the oncoprotein CagA, and the outer-membrane proteins (OMPs) BabA/B, OipA, and HopZ.

**Table 1.** Genotypes/allelic variants of virulence determinants encoding genes of the *H. pylori* strains included in this study, determined by whole genome sequencing.

*H. pylori*		Straingenotype											Patient		
Strain ID	Genome accession N°	*babA2*	*babB*	*babC*	*cagA*	*oipA*	*sabA*	*hopZ*	*homB*	*vacA*	*labA*	*hopQ*	Gender	Age (yr)	Outcome
771/99	SRR6906464	+	+	+	+	on	on	on	+ (1 copy)	s1	+	II	M	10	DU
417/02	SRR6906463	-	+	-	+	on	off	on	+ (2 copies)	-	-	I/II	M	1	DU
441/02	SRP064603*	+	+	+	+	on	on	off	-	s1^#^	+	I	F	9	DU
559/02	SRP064604*	+	+	-	+	on	off	on	+ (1 copy)	s1	+	I	M	15	DU
1776/05	SRP064606*	+	+	+	+	on	on	off	+ (1 copy)	s1	+	I	M	15	DU
1089/03	SRP071072*	-	+	+	+	on	off	on	+ (2 copies)	s1	-	I/II	M	10	DU
1152/02	JSUZ00000000**	+	+	+	+	on	off	on	+ (2 copies)	s1	-	I	M	10	DU
1846/05	SRP071062*	+	-	+	+	on	on	on	+ (2 copies)	s1	+	I	M	13	DU
1790/05	SRR6906462	+	+	+	+	off	on	off	+ (2 copies)	s1	+	I	M	6	DU
1198/04	SRP071060*	-	+	-	+	on	off	on	+ (2 copies)	s1	+	I	M	15	DU
499/02	SRP071064*	+	+	+	+	on	off	on	+ (2 copies)	s1	+	I	M	11	GU
517/99	SRR6906460	-	+	+	-	off	on	on	-	s2	+	II	M	14	NUD
892/99	SRR6906466	-	+	-	-	off	off	on	-	s2	+	I	F	14	NUD
207/99	SRP071066*	-	+	-	-	off	off	off	-	s2	+	I/II	F	7	NUD
173/00	SRP071065*	-	+	+	-	off	on	on	-	s2	+	II	M	14	NUD
655/99	SRP071069*	-	+	-	-	off	on	on	-	s2	+	II	M	11	NUD
1786/05	JSXW00000000**	+	+	-	-	off	on	on	-	s2	+	I/II	F	11	NUD
36/00	SRR6906461	-	+	-	-	off	on	off	+ (1 copy)	s2	+	I	M	9	NUD
565/99	SRR6906459	-	+	+	-	off	on	on	+ (1 copy)	s2	+	I	M	11	NUD
1500	SRR6906465	+	+	-	-	off	off	on	-	s2	+	I/II	F	13	NUD

M, male; F, female; DU, duodenal ulcer; GU, gastric ulcer; NUD, non-ulcer dyspepsia; +, positive for that gene; -, negative for that gene; ^#^, truncated s1 allele; * [44], ** [45]. The on and off frame status of the genes indicate if the gene is functional or not, respectively.

### At both neutral and acidic pH, PUD H. pylori strains bind better to mucins than NUD strains

Gastric mucins carry between 40 to100 different glycan structures resulting in a mucin glycosylation pattern that differs between individuals and also depends on health status [26]. We analyzed the binding levels of *H. pylori* strains isolated from children with NUD (n=9) or PUD (n=11), to gastric mucins with and without functional binding sites for the previously published *H. pylori* binding modes, BabA, SabA, LabA and the charge dependent mechanism at acidic pH [9-12]. The mucins tested were isolated from rhesus monkey normal gastric mucosa (Le^b^ negative ^(-)^, SLe^x^ positive ^(+)^, LacdiNAc^−^), human gastric tumor mucosa (Le^b+^, SLe^x+^, LacdiNAc^+,^ sulfate^+^), and human gastric normal mucosa (Le^b+^, SLe^x-^, LacdiNAc^+^) to provide an array of glycan structures (Table 2). Since *H. pylori* can infect both humans and monkeys [19], rhesus monkey mucins also represent a relevant carrier for glycans in this context.

**Table 2.** Origin, density, profile and glycan structures of the isolated mucin samples.

Mucinsamples	Density (g/L)	MUC5AC	MUC6	MUC2	MUC5B	Le^b^	SLe^x^	SLe^a^	α1,4-GlcNAc	LacdiNAc*	Acidic charge^*&^	A^#^	B^#^	H^#^
Human gastrictumor*^c^*	1.39 – 1.43	+	++	-	++	++	++	+	-	0.1	9.88	0.91	0	81.46
Human gastricnormal*^c^*	1.34 – 1.41	++	+	-	-	++	-	-	+	0.78	0	16.09	8.93	25.93
Monkeygastric normal*^e^*	1.38 – 1.43	+	++	-	-	-	+	+	++	0.1	1.1	0	47.8	4.4
Human gastric normal 3IS*^d,e^*	nd	nd	nd	nd	nd	+	-	-	-	7.5	10.7	22.4	1.1	60.8
Human gastric normal 4ID*^d,e^*	nd	-	+	nd	nd	-	+	+	+	16.9	16.8	2.9	0	0.4

The relative amount of mucins and glycan structures important for interactions with *H. pylori* were determined by ELISA and mass spectrometry. The relative presence or absence of each structure is indicated with ++ (above 75% of the highest assay value), + and – (OD_450_ less than 0.15 for antibodies giving a high signal and 0.1 for antibodies giving a low signal). Le^b^, Lewis b; SLe^x^, sialyl-Lewis x; SLe^a^, sialyl-Lewis a; nd, no data. * Relative abundance (%) determined by liquid chromatography tandem mass spectrometry. ^&^ Relative abundance of charged structures (NeuAc, NeuGc (present in the monkey normal mucin only), and sulfated structures (present in the human tumor mucin only) on mucins. ^#^ Relative abundance of ABH fucosylated antigens. *^c^* Data adapted from Skoog et al. [29], Linden et al. [39], and Jin et al. [26]. *^d^* Human gastric normal mucins used in the bacterial growth assays. *^e^* The complete list of identified structures and their relative abundances are available in the supplementary Table S1.

**Figure 1.**
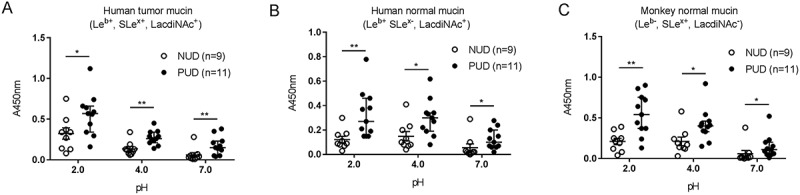
Binding of *H. pylori* to gastric mucins. Binding of *H. pylori* strains isolated from children with non-ulcer dyspepsia (NUD, n=9) or with peptic ulcer disease (PUD, n=11) to mucins isolated from human tumor (A), human normal (B) and monkey normal (C) gastric tissue. Results are expressed as the median with interquartile range, after subtraction of the background signal (no mucin) at each pH. Mann-Whitney U test comparing the binding of NUD vs PUD *H. pylori* strains to mucins at each pH, * *p* ≤ 0.05, ** *p* ≤ 0.01.

The adhesion experiments were performed at pH 2.0, pH 4.0, and pH 7.0, representing the pH gradient found in the gastric mucus layer, with an acidic pH towards the gastric lumen and a neutral pH towards the epithelium. The results showed that both NUD and PUD *H. pylori* strains bound to human and monkey mucins independently of their Le^b^, SLe^x^, LacdiNAc and acidity (sialic acid) status (Figure 1). Absence of binding was observed mainly at pH 7: two PUD and seven NUD strains lacked binding to human mucins, while seven NUD and one PUD strain did not bind to monkey mucins.

Binding of the *H. pylori* strains isolated from PUD patients was increased compared to the binding of NUD strains to all mucins at both acidic and neutral pH, with higher binding activity at pH 2 than at pH 7 (*p*≤0.05, Figure 1(a-c)). Based on the strain genotype (*babA, sabA* and *labA* status) and mucin glycosylation, a similar proportion of the strains are expected to bind to mucins among the PUD and NUD strains at pH 7: 8/11 PUD vs 6/9 NUD would theoretically be expected to bind to the mucin from the human gastric tumor mucosa (Le^b+^, SLe^x+^, LacdiNAc^+^, sulfate^+^), 9/11 PUD vs 9/9 NUD to the mucin from the human gastric normal mucosa (Le^b+^, SLe^x-^, LacdiNAc^+^) and 5/11 PUD vs 5/9 NUD to the mucin from the monkey normal gastric mucosa (Le^b-^, SLe^x+^, LacdiNAc^−^, Table 3). Of the strains expected to bind to the human gastric tumor mucin 7/8 PUD and 3/6 NUD strains matched the predicted binding at pH 7. Similarly, 8/9 PUD and 2/9 NUD strains matched the expected binding to mucins from human gastric normal mucosa at pH 7. Binding of *H. pylori* to the monkey mucins was shown in all five PUD strains expected to bind, and in 2/5 NUD strains at pH 7. Additionally, 1/3 PUD and 3/3 NUD strains lacking binding to the human tumor mucins matched the predicted no binding at pH 7. No matches between non-binding NUD and PUD strains and predicted no binding to human normal mucin were observed at pH 7, while four out of four NUD and one out of six PUD strains matched the predicted no binding to monkey mucins. Strain genotype can thus not fully predict binding or binding amplitude to mucins. This is likely due to related glycans that can act as ligands for the adhesins in a manner that varies between strains, and that the topography of the glycans can result in differences in accessibility between mucins for binding.

**Table 3.** Predicted pH where binding is mainly expected based on presence of the BabA, SabA and LabA adhesins in the genome of the *H. pylori* strains (Table 1) and glycans known to confer binding identified on the mucins (Table 2).

*H. pylori*		Mucintype and mucin glycans				
Strain	Patient	Human gastrictumor	Human gastric normal	Monkeygastric normal	Human gastric normal 3IS	Human gastric normal 4ID
ID	outcome	(Le^b^, SLe^a/x^, S)	(Le^b^, LacdiNAc)	(SLe^a/x^)	(Le^b^, LacdiNAc)	(SLe^a/x^, LacdiNAc)
771/99	PUD	pH 2 and 7	pH 7	pH 7	pH 7	pH 7
417/02	PUD	pH 2	-	-	-	-
441/02	PUD	pH 2 and 7	pH 7	pH 7	pH 7	pH 7
559/02	PUD	pH 2 and 7	pH 7	-	pH 7	pH 7
1776/05	PUD	pH 2 and 7	pH 7	pH 7	pH 7	pH 7
1089/03	PUD	pH 2	-	-	-	-
1152/02	PUD	pH 2 and 7	pH 7	-	pH 7	-
1846/05	PUD	pH 2 and 7	pH 7	pH 7	pH 7	pH 7
1790/05	PUD	pH 2 and 7	pH 7	pH 7	pH 7	pH 7
1198/04	PUD	pH 2	pH 7	-	pH 7	pH 7
499/02	PUD	pH 2 and 7	pH 7	-	pH 7	pH 7
517/99	NUD	pH 2	pH 7	-	pH 7	pH 7
892/99	NUD	pH 2	pH 7	-	pH 7	pH 7
207/99	NUD	pH 2	pH 7	-	pH 7	pH 7
173/00	NUD	pH 2 and 7	pH 7	pH 7	pH 7	pH 7
655/99	NUD	pH 2 and 7	pH 7	pH 7	pH 7	pH 7
1786/05	NUD	pH 2 and 7	pH 7	pH 7	pH 7	pH 7
36/00	NUD	pH 2 and 7	pH 7	pH 7	pH 7	pH 7
565/99	NUD	pH 2 and 7	pH 7	pH 7	pH 7	pH 7
1500	NUD	pH 2 and 7	pH 7	-	pH 7	pH 7

Only pH 2 and 7 were included since the pH dependency slope for the binding modes can vary between strains. Although the monkey gastric normal sample is negative for Le^b^, it carries other fucosylated structures that have potential for interacting with BabA. However, in the table we only included Le^b^ as BabA ligand as this is expected to be the main structure conferring binding. Similarly, only the human gastric tumor sample is listed for expected theoretical interaction with *H. pylori* at pH 2, as this sample carries sulfated structures in addition to sialylated ones, whereas the other samples carry sialylated structures whereof only 11% are expected to remain negatively charged at pH2. S, Sulfate; -, absence of known ligand-adhesion interactions; NUD, non-ulcer dyspepsia; PUD, peptic ulcer disease.

Binding at pH 2 is mainly expected to be conferred via a charge dependent mechanism [27]. In line with this, the highest binding avidity was detected to the mucins from human gastric tumor mucosa followed by mucins from the monkey normal gastric mucosa and then the human gastric normal mucosa. Altogether, the results depict binding properties that differ between NUD and PUD for all mucins at all pHs (*p* < 0.05), and that increase in an acidic environment.

### Increased binding of gastric mucins to PUD H. pylori baba positive strains compared to NUD baba positive strains at acidic pH

**Figure 2.**
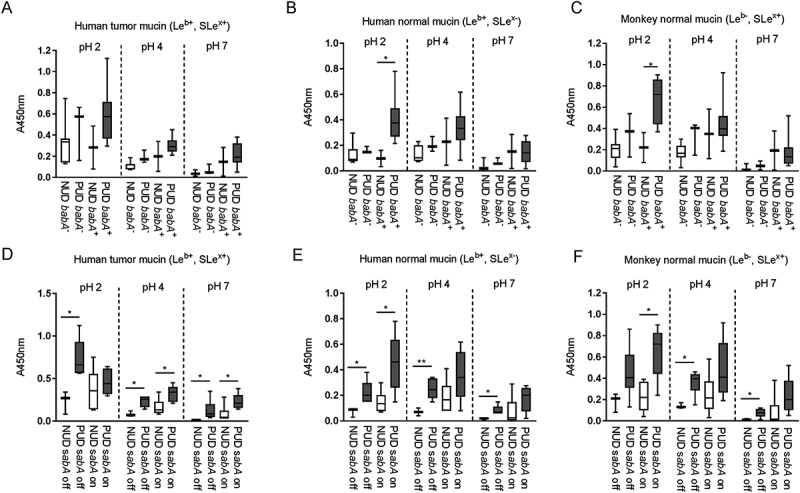
Binding of *H. pylori* pediatric strains to gastric mucins according to their *babA* and *sabA* genotype. A-C) Binding of NUD and PUD *H. pylori babA*^+^ and *babA*^−^ strains (NUD *babA*^−^, n = 7; PUD *babA*^−^, n = 3; NUD *babA*^+^, n = 2; PUD *babA*^+^, n = 8) to mucins isolated from human tumor (A), human normal (B) and monkey normal (C) gastric tissue. D-F) Binding of NUD and PUD *H. pylori sabA*^+^ and *sabA*^−^ strains (NUD *sabA* off, n = 3; PUD *sabA* off, n = 6; NUD *sabA* on, n = 6; PUD *sabA* on, n = 5) to mucins isolated from human tumor (D), human normal (E) and monkey normal (F) gastric tissue. Results are expressed as minimum, first quartile, median, third quartile, and maximum in a box and whisker plot, after subtraction of the background signal (no mucin) at each pH. Mann-Whitney U test comparing binding to mucins of NUD vs PUD strains with the same genotype, * *p* ≤ 0.05, ** *p* ≤ 0.01.

BabA and SabA are well studied *H. pylori* outer membrane proteins that mediate adhesion to the gastric mucosa, and the *babA*^+^ genotype has been associated with ulcer disease [5]. We characterized the binding ability of NUD and PUD strains to gastric mucins according to their *babA* and *sabA* status to assess their role in binding. The *babA*^+^ and *sabA* “on” genotypes were present in both NUD and PUD *H. pylori* strains, with *babA*^+^ strains predominantly distributed within the PUD group (Table 2). At neutral pH, adhesion of *babA*^+^ strains was higher than that of *babA*^−^ strains and the level of binding of NUD and PUD *babA*^+^ strains to mucins was similar (Figure 2(a-c)).Thus, differences in binding between NUD and PUD strains at neutral pH may be largely due to the higher proportion of *babA*^+^ strains in the latter group. At pH 2 however, the PUD *babA*^+^ strains displayed higher binding to mucins isolated from human and monkey normal gastric tissue compared to NUD *babA*^+^ and *babA*^−^ strains of both PUD and NUD origin (*p* = 0.05; Figure 2(b,c)), indicating the PUD *babA* has different adhesion characteristics at acidic pH than NUD *babA*. Contrarily, the *sabA* status does not appear to be a factor responsible for differences in mucin binding between PUD and NUD strains, as PUD *H. pylori* strains bound more avidly to mucins compared to the NUD strains regardless of this genotype (Figure 2(d-f)).

### Increased le^b^ binding among baba positive PUD H. pylori strains compared to NUD strains at neutral, but not acidic ph

**Figure 3.**
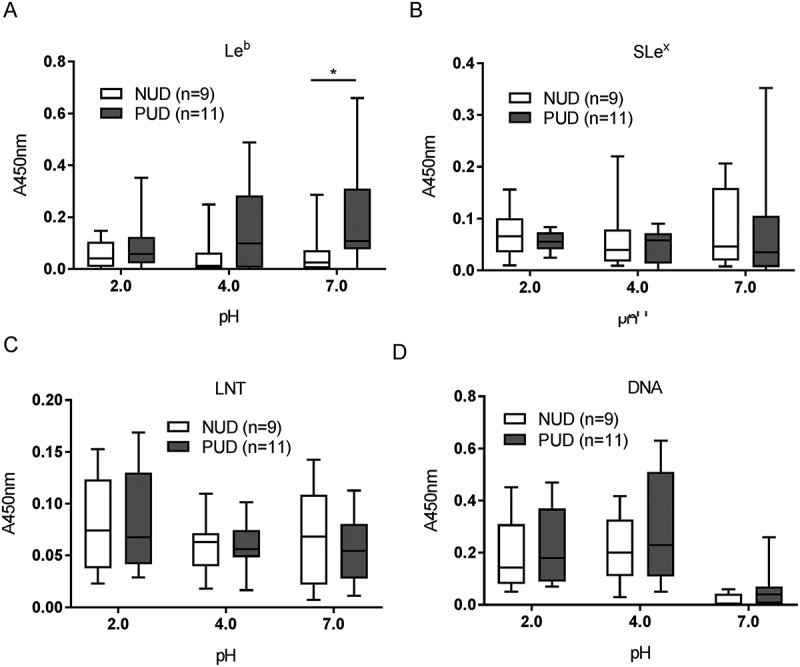
Binding of *H. pylori* to glycoconjugates and DNA. Binding of NUD (n = 9) and PUD (n = 11) *H. pylori* pediatric strains to Le^b^ (A), SLe^x^ (B), LNT (C), and DNA (D). Results are expressed as minimum, first quartile, median, third quartile, and maximum in a box and whisker plot, after subtraction of the background signal (no mucin) at each pH. Mann-Whitney U test comparing the binding of NUD vs PUD *H. pylori* strains to glycoconjugates and DNA at each pH, * *p* ≤ 0.05.

In order to elucidate the molecular mechanism mediating the difference in binding between NUD and PUD *H. pylori* strains to mucins, we investigated the five *H. pylori* binding modes known to be involved in binding to gastric mucins (Le^b^, SLe^x^, LNT, LacdiNAc and charge dependent binding) at pH 2.0, 4.0, and 7.0. Differences in adhesion between NUD and PUD *H. pylori* strains were observed to the Le^b^ moiety, recognized by the BabA adhesin at neutral pH (Figure 3(a)). The majority of the *babA*^+^ strains had a strong adhesion to Le^b^ (referred to as Le^b^ high binders) except for the PUD strain 1776/05, which presented a binding level similar to the NUD and PUD *babA*^−^ strains (Le^b^ low binders, Figure 5(a)).

**Figure 4.**
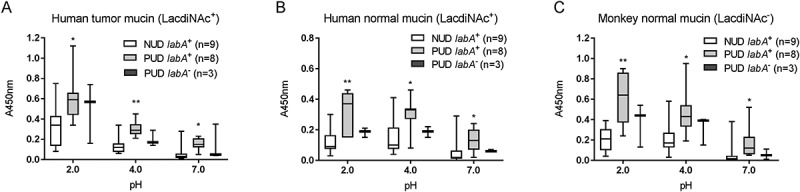
Binding of *H. pylori* to mucins according to *labA* genotype. Binding of NUD (n = 9) and PUD (n = 11) *H. pylori* strains to mucins isolated from human tumor (a), human normal (b), and monkey normal (c) gastric tissue. Results are expressed as minimum, first quartile, median, third quartile, and maximum in a box and whisker plot. Kruskall-Wallis test with Dunn’s correction for multiple comparisons, * *p* ≤ 0.05 and ** *p* ≤ 0.01 compared to NUD *labA^+.^*

**Figure 5.**
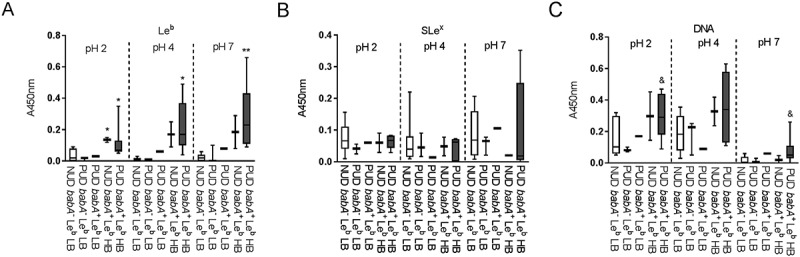
Binding of *H. pylori* strains to glycoconjugates and DNA according to their *babA* genotype and avidity for Le^b^. Binding of NUD and PUD *H. pylori* strains to Le^b^ (a), SLe^x^ (b), and DNA (C). NUD *babA*^−^ Le^b^ low binder (LB), n = 7; PUD *babA*^−^ Le^b^ low binder (LB), n = 3; PUD *babA*^+^ Le^b^ low binder (LB), n = 1; NUD *babA*^+^ Le^b^ high binder (HB), n = 2; PUD *babA*^+^ Le^b^ high binder (HB), n = 7. Results are expressed as minimum, first quartile, median, third quartile, and maximum in a box and whisker plot. Kruskall-Wallis test with Dunn’s correction for multiple comparisons, * *p* ≤ 0.05 and ** *p* ≤ 0.01 compared to NUD *babA*^−^ Le^b^ low binder. Mann-Whitney U test, ^&^ *p* ≤ 0.05 compared to NUD and PUD *babA*^−^ Le^b^ low binders.

The overall binding to the SLe^x^ antigen recognized by SabA, and LNT was of low avidity and no clear trends were identified regarding pH dependency or differences between NUD and PUD groups (Figure 3(b-c)). Furthermore, the NUD/PUD origin and the *babA* status of the strains were not relevant in binding to SLe^x^ (Figure 5(b)). LacdiNAc carried by gastric mucins has been described as a target for *H. pylori* adherence to the gastric mucosa via the LabA adhesion [12]. Although the purified monkey mucin was negative for LacdiNAc and the human mucins carried the LacdiNAc motif (Table 1), the binding level among NUD and PUD strains to mucins was similar, and overall binding level to mucins was not proportional to the LacdiNAc content of the mucins (Figure 4, Table 1). Furthermore, the majority of strains were positive for LabA, and LabA was present among all NUD strains (Table 2). Consequently, the results do not suggest a role of LacdiNAc, SLe^x^ or LNT as determinants relevant for the difference in binding of *H. pylori* NUD and PUD strains to gastric mucins.

Binding to DNA was used as a proxy for binding to charged molecules via the charge/low pH mechanism (such as sialylation and sulfation present on mucins), as DNA carries a high density of negative charge. *H.pylori* binding to DNA was more pronounced at acidic than neutral pH, although similar between NUD and PUD strains (Figure 3(d)). Surprisingly, PUD *babA*^+^ strains with high binding ability to Le^b^ bound more to DNA at pH 2 and 7 compared to PUD and NUD *babA*^−^ strains (*p* ≤ 0.05, Figure 5(c)).

### Attenuated binding of PUD baba-knockout mutants to mucins and le^b^ at acidic and neutral ph, and to sle^x^ and DNA at acidic pH

**Figure 6.**
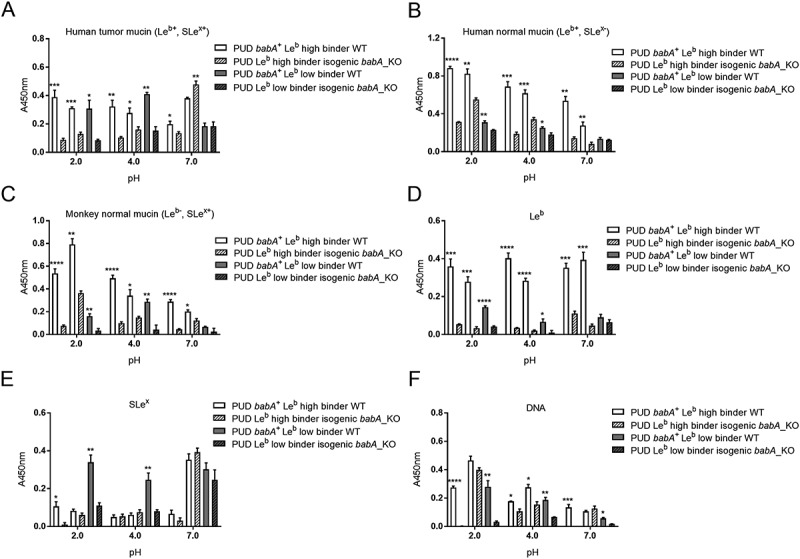
Binding of NUD and PUD *H. pylori* wild type and isogenic *babA*-knockout mutant strains to mucins isolated from human tumor (A), human normal (B) and monkey normal (C) gastric tissue, Le^b^ (D), SLe^x^ (E), and DNA (F). Data is grouped according to strain *babA* status and binding ability to Le^b^: PUD *babA*^+^ Le^b^ high binder WT (n = 2, 559/02 and 441/02), PUD Le^b^ high binder isogenic *babA*_KO (n = 2, 559/02 and 441/02 *babA*_KO), PUD *babA*^+^ Le^b^ low binder WT (n = 1, 1776/05), and PUD Le^b^ low binder isogenic *babA*_KO (n = 1, 1776/05 *babA*_KO). Results are expressed as the mean of technical replicates ± SEM, after subtraction of the background signal (no mucin) at each pH. Student’s *t*-test comparing the binding of the WT strain vs their respective isogenic *babA* KO strain, * *p* ≤ 0.05, ** *p* ≤ 0.01, *** *p* ≤ 0.001, **** *p* ≤ 0.0001.

To determine the functional role of *babA* in NUD and PUD *H. pylori* adhesion, we analyzed the binding ability of *H. pylori babA*-knockout mutant strains to human and monkey mucins, Le^b^, SLe^x^, and DNA compared to their isogenic wild-type strains (Figure 6). For this, we selected three *babA*^+^ PUD strains, whereof two were Le^b^ high binders and one was a Le^b^ low binder, and all three had a low pH dependency in their Le^b^ binding (i.e. binding to Le^b^ was similar at pH 2 and 7). Removal of *babA* reduced the adherence of *babA*^+^/Le^b^ high binding wild-type strains (n = 2) to all three mucins and to the Le^b^ glycoconjugateat all pHs (Figure 6(a-d)). An exception to this was that binding of the *sabA* and *babA* positive strain 441/02 to the human tumor mucin (Le^b+^ and SLe^x+^) at pH 7 instead increased (Figure 6(a)): we have previously observed that other adhesion modes can gain after removal of *babA* [28]. In line with this concept, there was a trend towards an increase in binding to SLe^x^ too with this strain at pH 7 (Figure 6(e)). Adhesion of the *babA*^+^/Le^b^ low binding strain (n =1) to human and monkey mucins and to Le^b^ was attenuated at pH 2 and 4 after knocking out *babA*, but the effect was not statistically significant at pH 7 (Figure 6(a-d)). Two out of three *babA* deletion mutants exhibited reduced binding to SLe^x^ and DNA at acidic pH compared to their isogenic wild-type strains (*p*<0.05), and a similar trend was observed for the third strain (SLe^x^, *p*= 0.16 and DNA, *p* = 0.22, Figure 6(e,f)). This indicates a role for BabA in charge dependent adhesion at acidic pH.

### No indications that the homB, oipA, and cagA virulence genes play a role in the differential binding of NUD and PUD strains to mucins, synthetic glycoconjugates and DNA

**Figure 7.**
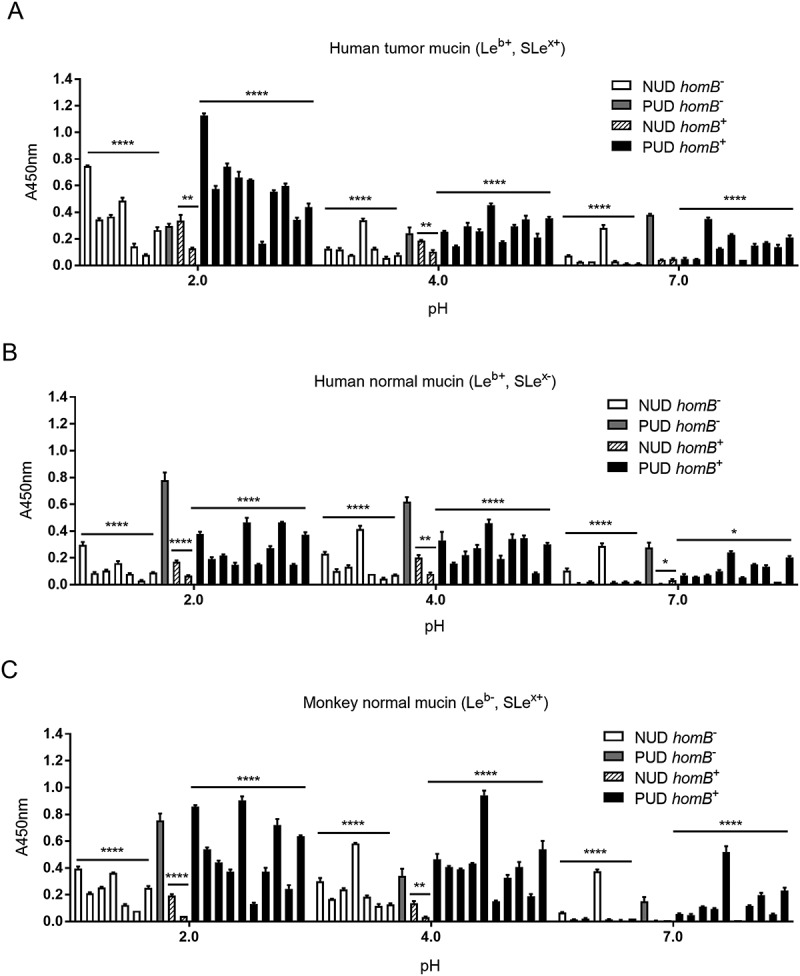
Binding of *H. pylori* to mucins grouped according to their *homB* genotype. Binding of *H. pylori* strains isolated from children with non-ulcer dyspepsia (NUD, n = 9) or with peptic ulcer disease (PUD, n = 11) to mucins isolated from human tumor (A), human normal (B) and monkey normal (C) gastric tissue according to their *homB* status. Results are expressed as the average of technical replicates ± SEM, after subtraction of the background signal (no mucin) at each pH. Overall One-way ANOVA comparing the binding of *H. pylori* strains to mucins within each group (* *p* ≤ 0.05, ** *p* ≤ 0.01, **** *p* ≤ 0.0001), demonstrating that statistically significant differences are present between the strains within each group,e.g. within NUD *homB*^−^. However, there are no differences between NUD and PUD derived strains.

**Figure 8.**
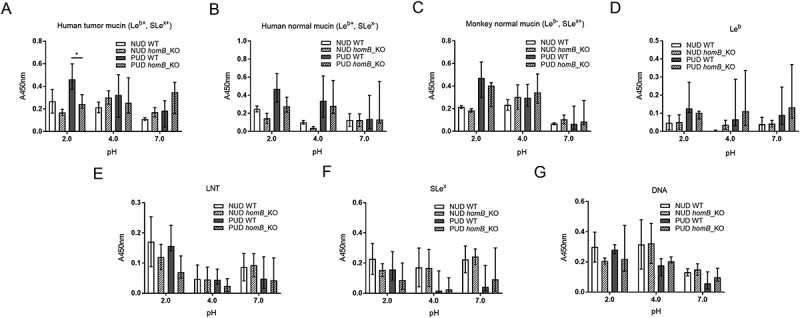
Binding of *H. pylori* wild-type and isogenic *homB-*knockout mutant strains to mucins, glycoconjugates, and DNA. Binding of *H. pylori* wild-type (WT) strains isolated from children with non-ulcer dyspepsia (NUD, n = 2) or with peptic ulcer disease (PUD, n = 5) and their isogenic *homB-*knockout (KO) mutant strains to mucins isolated from human tumor (A), human normal (B) and monkey normal (C) gastric tissue, to Le^b^ (D), LNT (E), and SLe^x^ (F) glycoconjugates, and to DNA (G). Results are expressed as the median with interquartile range, after subtraction of the background signal (no mucin) at each pH. Mann-Whitney U test comparing the binding of the WT strain vs their respective isogenic *homB* KO strain, * *p* ≤ 0.05.

*HomB* positive ^(+)^ *H. pylori* clinical isolates have been associated with PUD in children [4], and in line with this study the proportion of *homB*^+^ strains was higher among the *H. pylori* PUD than NUD strains (Table 1). *HomB*^+^ strains have been suggested to bind better to a human gastric epithelial cancer cell line (AGS) than *homB*^−^ strains [4]. However, there was no association between the *homB*genotype of the NUD and PUD *H. pylori* strains and binding to mucins, Le^b^, SLe^x^, or LNT glycoconjugates, or DNA (marker for acidic charge) at any of the pH tested, and binding varied between strains (Figure 7(a-c)). Furthermore, adhesion of the *homB-*knockout mutant strains to mucins, glycoconjugates and DNA was not different from their isogenic wild-type strains (Figure 8(a-g)), except lower binding of the PUD *homB*-knockout mutants to the human gastric tumor mucin at pH 2 (*p* = 0.02, Figure 8(a)). In a similar manner, there was no clear trend in binding of the NUD and PUD *H. pylori* strains to mucins according to their *oipA* genotype (data not shown). As a result of all PUD strains being *cagA*^+^ and all NUD strains *cagA*^−^ (Table 1), binding to mucins of PUD *cagA*^+^ *H. pylori* strains was higher than the NUD group at all pH (data not shown). However, the *cagA* gene product plays a role after bacterial attachment to gastric epithelial cells and is injected into cells via the type IV secretion system; therefore, a causal association between this genotype and binding to gastric mucins is unlikely. Overall, no major effects of *homB* and *oipA* genotypes on *H. pylori* adhesion to mucins were identified.

### The le^b^carrying mucin and le^b^ glycoconjugate inhibit growth of PUD H. pylori babA positive strains at ph 5 and 7, whereas the ɑ1,4-GlcNAc positive mucin has an inhibitory effect on both NUD and PUD strains

**Figure 9.**
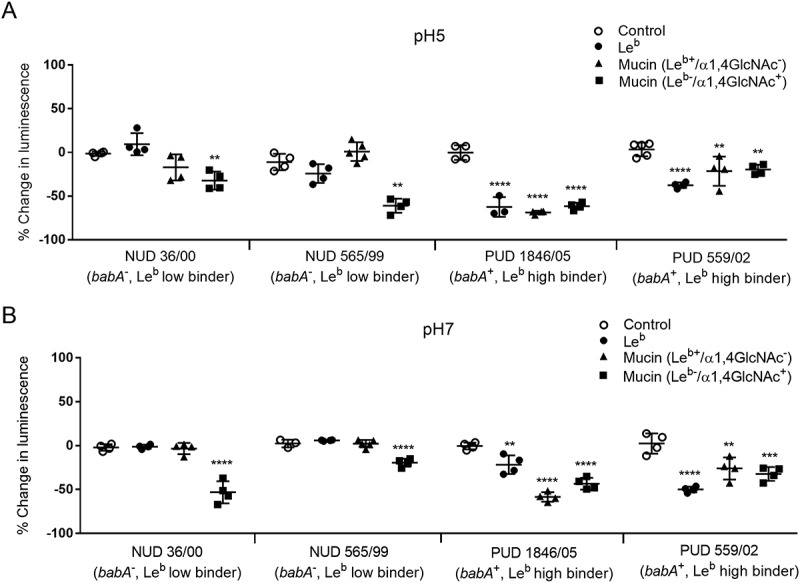
*H. pylori* proliferation in the presence of mucins and Le^b^. Effect of gastric mucins and Le^b^ on the growth of NUD and PUD *H. pylori* pediatric strains at pH 5 (A) and 7 (B). Results are expressed as the mean percent difference of the luminescent signal compared to the proliferation control ± SEM. One-Way ANOVA with Dunnett’s correction for multiple comparisons, ** *p* ≤ 0.01, **** *p* ≤ 0.0001.

Mucins can have an inhibitory or stimulatory effect on *H. pylori* proliferation mainly depending on mucin glycosylation and binding ability [29]. As differences were observed in adhesion of NUD and PUD strains to mucins and Le^b^ we studied the effect of purified gastric mucinsand the Le^b^ glycoconjugate on *H. pylori* proliferation. The assays were performed at pH 7 and 5. Two NUD *H. pylori* strains (36/00 and 565/99) both *babA*^−^ and low Le^b^ binders, and two *babA*^+^ PUD strains (559/02 and 1846/05) with high binding ability to Le^b^ were co-cultured separately with two differentially glycosylated human gastric mucins (Le^b+^/ɑ1,4-GlcNAc^−^and Le^b-^/ɑ1,4-GlcNAc^+^) and the Le^b^ glycoconjugate. The reducing potential of viable cells was measured over time by a luciferase based kit in which the luminescent signal is proportional to the number of viable cells in culture. Similarly to a report on the inhibitory effect of Le^b^ on *H. pylori* proliferation at neutral pH [28], our results show growth inhibition of the PUD *babA*^+^ *H. pylori* strains in response to the Le^b^ glycoconjugate and Le^b^ positive mucin at pH 7 and 5 (*p* ≤ 0.01, Figure 9(a,b)). The Le^b^ glycoconjugate and Le^b^ carrying mucin had no effect on the proliferation of NUD *babA*^−^ strains (Figure 9(a,b)), suggesting that the inhibitory effect of Le^b^ on *H. pylori* is BabA dependent. Previous studies have demonstrated antimicrobial activity of mucins carrying terminal ɑ1,4-GlcNAc [28,30], and in line with this, a mucin carrying ɑ1,4-GlcNAc inhibited growth of both NUD and PUD *H. pylori* strains regardless of pH (*p* ≤ 0.01, Figure 9(a,b)).

## Discussion

In this study, we characterized the binding ability and adhesion modes of *H. pylori* strains isolated from pediatric patients with NUD and PUD to gastric mucins at acidic and neutral pH. Our results showed that pediatric *H. pylori* strains isolated from children with PUD display an increased binding activity to gastric mucins at both neutral and acidic pH compared to NUD strains. The PUD *H. pylori babA* positive genotype was associated with increased adhesion to Le^b^ at neutral pH and via the charge dependent binding mode at acidic pH, suggesting a role for BabA separate from its specific blood group binding activity. Furthermore, the reduced adherence of PUD *babA*-knockout mutants to mucins, DNA and Le^b^ indicate a functional role of BabA in the adherence of pediatric ulcerogenic strains both under neutral and acidic conditions.

*H. pylori* colonization of the stomach generally occurs during early childhood [31]. Although gastric ulcers seldom occur in the pediatric population, when present, they develop shortly after infection [25]. Studies have pointed at enhanced virulence features among *H. pylori* ulcerogenic strains isolated from children compared to non-ulcerogenic strains, resulting in impairment of gastric cell viability with cytoskeletal damage accompanied by decreased intracellular mucin content *in vitro* [25]. Ulcerogenic pediatric strains share a similar proteome profile and several putative virulence genes, such as *cagA, vacAs1, oipA* on status, *homB* and *jhp562*, have been related with ulcer disease in children [4,6,25,32,33]. It has been suggested that the pediatric *H. pylori* strains carrying the triple genotype *cagA, jhp562*, and *homB*are clinically relevant in PUD outcome [33]. In our study, nearly all PUD strains carried the *vacA*s*1, cagA, oipA* on status and *homB* genes, contrary to the virulence profile of the NUD strains. However, there were no indications that the aforementioned genes play a biological role in the differential adhesion of pediatric ulcerogenic strains to gastric mucins. The clinical relevance of *H. pylori* adhesins, such as BabA2, and Lewis antigens in the pathogenesis of ulcer disease has been reported [5]. The *BabA2* genotype was predominant among the screened pediatric *H. pylori* PUD strains; furthermore, the *babA2* positive ulcerogenic strains concomitantly displayed increased adhesion ability to gastric mucins, pointing towards the biological relevance of *babA* in gastric colonization and pediatric ulcer disease development.

Bacterial adherence to secreted mucins depends both on the host’s mucin glycosylation and on adhesins expressed by the bacteria. Gastric mucins carry a diversity of carbohydrate structures, some of which serve as binding sites for *H. pylori*. Mucin expression and glycosylation (Le^a^, Le^b^, Le^x^, SLe^x^ and SLe^a^) has been shown to be similar in the stomach of uninfected children and adults [23]. In an American cohort, pediatric *H. pylori* infection was not associated with ectopic MUC6 or MUC2 expression [23],whereas MUC2 antibody reactivity increased with age among pediatric samples in an Asian cohort [34]. Tissue sections from *H. pylori* infected pediatric patients contain a lower proportion of mucus producing cells than non-infected tissues due to infiltrating immune cells, and the tall epithelial cells loaded with mucus are often shorter/flatter with less mucus in the mucin thecae during infection [23]. This is likely the reason why the proportion of the tissue that stained positive for MUC5AC was lower in infected pediatric samples [34]. Gastric mucins secreted from the superficial mucosa can prevent *H. pylori* binding to the epithelial surface [21]. Similar to rhesus monkeys, Le^b^ positive children had a lower *H. pylori* density than Le^b^ negative children, highlighting a role of secretor and Lewis status in resistance to infection [19,23]. However, among children with PUD, the proportion that are Le^b^ positive is similar to that of the general population [23]. Mucin glycosylation can also affect *H. pylori* expression of virulence genes and growth by either having a proliferative or inhibitory effect [28,29]. *H. pylori* BabA dependent binding to human gastric mucins has been shown to inhibit proliferation due to bacterial aggregation and mucins carrying the ɑ1,4-GlcNAc terminal structure have antimicrobial activity [28,30]. Similarly, gastric mucins decorated with Le^b^ as well as the synthetic Le^b^ structure repressed the growth of *babA* positive strains and mucins carrying ɑ1,4-GlcNAc repressed growth of all strains regardless of NUD/PUD origin and *babA* status.

To our knowledge, there are no previous reports on the binding ability and adhesion modes of *H. pylori* strains isolated from children to gastric mucins. Since the gastric mucus layer provides an acidic to neutral pH gradient, we considered relevant to study the effect of pH on pediatric *H. pylori* adhesion to mucins. Our observations showed that both at neutral and acidic pH *H. pylori* PUD strains had the intrinsic ability to bind better to all mucins compared to the NUD strains, with enhanced binding properties in acidic conditions. In addition to that a high proportion of PUD strains are positive for *babA*, the increase in binding at acidic pH was associated to *babA* expression, as PUD *H. pylori babA*^+^ strains bound considerably more to gastric mucins than NUD *babA*^±^ strains. Although the pH optimum in binding to Le^b^ was at pH 7 in line with published studies showing that the specific blood group binding activity of BabA has a neutral pH optimum [27,35], mucin binding at neutral pH was considerably lower than that observed at acidic pH. Adhesion at acidic pH was twice as high to the mucins that carried charged structures (such as SLe^x^) compared to the mucins lacking SLe^x^, but this effect was not dependent on the presence of SabA. BabA from different strains have been shown to vary in which fucosylated blood group related structures they preferentially bind to, both to individual structures and when present on mucins [9,10]. Furthermore, BabA differ in pH sensitivity among strains, with strains isolated from corpus loosing Le^b^ binding activity at a lower pH than strains isolated from antrum [35]. In line with these results, high binding level to mucins remained at acidic pH among the PUD strains: for some strains BabA dependent binding to Le^b^ remained high whereas for others BabA dependent binding to charged glycan structures was a prominent feature. Peptic ulcers are associated with a more acidic pH [36], suggesting that the pathogen adapts to a more acidic environment. Hence, at both acidic and neutral pH pediatric ulcerogenic *babA*^+^ strains displayed adhesion properties distinct from non-ulcerogenic strains. The functionality of *babA* was further supported by loss in adherence to mucins and Le^b^ after removal of *babA* at both acidic and neutral pH. Additionally, at acidic pH removal of *babA* decreased *H. pylori* binding to SLe^x^ and DNA in two out of three strains, suggesting that *babA* can be involved in charge dependent adhesion.

**Figure 10.**
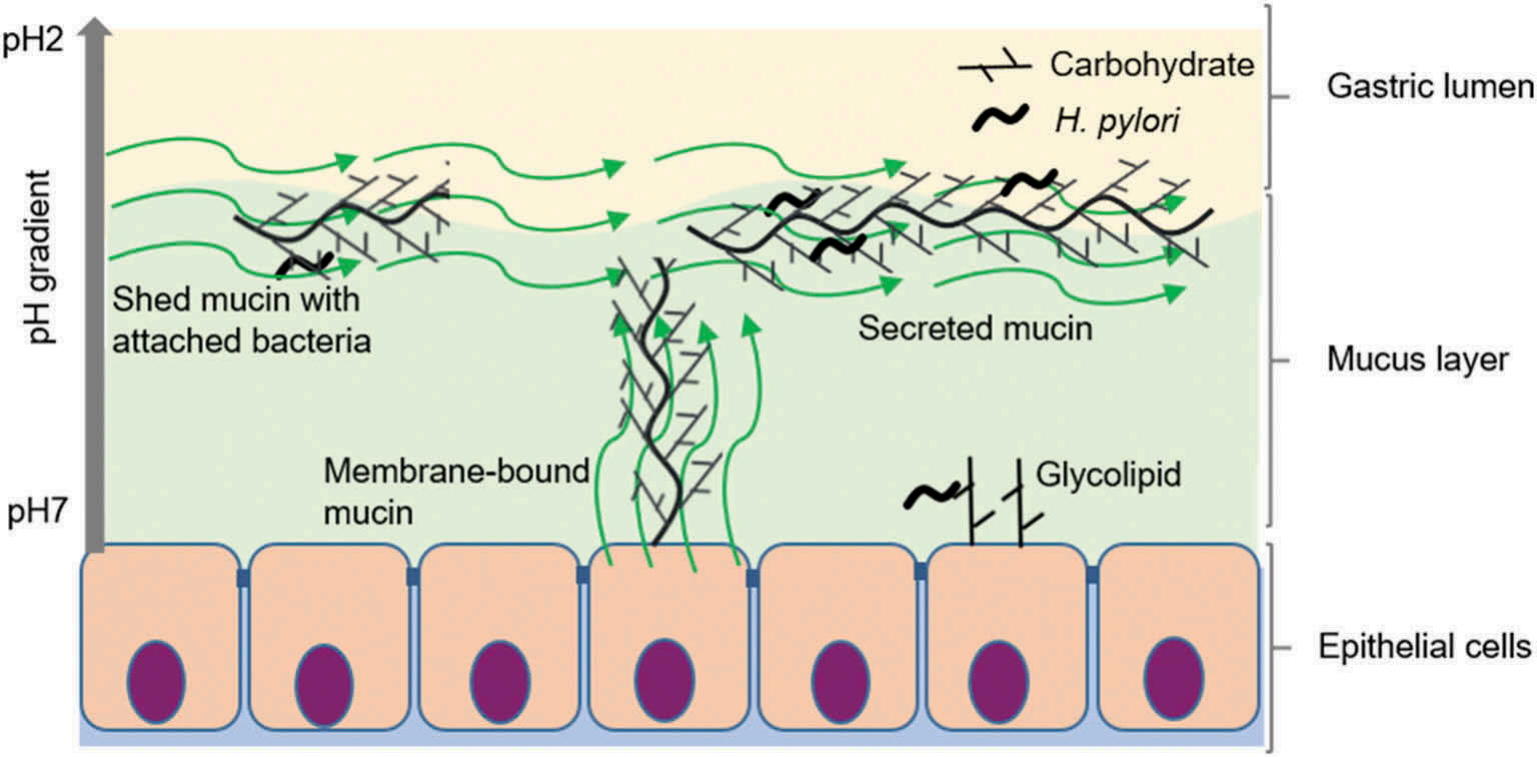
Consequences of *H. pylori* binding to secreted mucins, membrane bound mucins and glycolipids. Depending on the genotype of the infecting strain as well as the glycans expressed by the host (depends on inflammatory status and host genotype), *H. pylori* can bind to glycans on glycosphingolipids, membrane-bound mucins and secreted mucins at neutral pH close to the epithelial cells as well as to mucins in the mucus layer or lumen at acidic pH. The mucins constantly flow away from the epithelial surface, are shed into the lumen and removed from the stomach with the gastric emptying. Please note that components in the figure are not drawn to scale: i.e. in the body, the mucus layer is thicker and *H. pylori* smaller compared to other components in the figure.

Although PUD strains had a higher level of binding to mucins than NUD strains, we do not believe that having mucins that bind to pathogens is negative to the host. On the contrary, both rhesus monkeys and children that produce mucins that bind well to *H. pylori* have lower gastritis and pathogen burden in their stomachs [19,23]. Furthermore, mice lacking the Muc1 mucin are more susceptible to infection by *H. pylori* [37]. The pathogenicity decreasing effect of mucins is likely due to a combination of mucins binding and disseminating the pathogen and by that mucin binding can decrease the growth of the pathogen [19,28]. Mucins can carry similar glyco-epitopes as glycolipids, and act as a decoy for the intimate adherence conferred by *Helicobacter* adhesion to glycolipids in the gastric epithelium [9,21].The higher binding of PUD strains to mucins shown here, most likely reflects a higher avidity for fucosylated and sialylated glycolipid targets on the epithelial cell surface. Although the pH at the epithelial surface is close to neutral under healthy conditions, increased acid production or damaged mucus layer has potential to decrease the pH at the epithelial surface. Thus, the higher binding to mucins by PUD strains in combination with previous studies demonstrating a host protective role for mucin binding [19,21,23,38], suggest that PUD strains have a higher fitness for binding to the host although the host also produces binding decoys on mucins and mucin-bound bacteria are removed and disseminated with the shedding of the mucus (Figure 10).

In conclusion, *H. pylori* isolated from pediatric patients with PUD bind to mucins with higher avidity at both acidic and neutral pH than those isolated from children with NUD. This higher avidity is caused by a combination of higher prevalence of BabA and BabA binding to charged structures (i.e. sulfated and sialic acid containing structures) at acidic pH.

## Materials and methods

### Ethics

Three human samples were collected after informed written consent and in accordance with approval of the local ethics committee (Lund University Hospital, Lund, and Regionalaetikprövningsnämnden I Göteborg, Sweden). The fourth specimen was from a tumor sample from our well characterized mucin library, collected in 1983 at the IMIM-Hospital del Mar, Barcelona, Spain (before the hospital had an ethics committee). The rhesus monkey samples were also from our mucin library, collected in conjunction with a previous study [39]. These samples were collected in accordance with approval of the Uniformed Services University of the Health Sciences animal care and use committee, USA).

### Isolation of mucins

The main part of the study was performed on mucins isolated from two human gastric specimens and one rhesus monkey gastric tissue. One human specimen was from a gastric adenocarcinoma tumor (intestinal type, hereafter referred to as human tumor sample) and the other one from macroscopically normal antral mucosa of a tumor-affected stomach (hereafter referred to as human normal sample), as evaluated by a clinical pathologist. The rhesus monkey sample was positive for *H. suis* but was otherwise normal (gastritis score < 1 according to the Sydney system) [39]. The isolated human mucins have been previously characterized in detail [26,29,39]. We also screened three rhesus monkeys gastric mucins from our archived mucin library using mass spectrometry (results in supplementary table S1) and selected one negative for Le^b^ and lacdiNAc to complement the glycan repertoir of the human mucins [26]. Additionally, two mucins (3IS: Le^b^ positive/ɑ1,4-GlcNAc negative, and 4ID: Le^b^ negative/ɑ1,4-GlcNAc positive) were isolated from human normal gastric tissue obtained from obese patients undergoing bariatric surgery and were used in bacterial proliferation assays. The mucins were isolated from tissue specimens after rinsing with phosphate-buffered saline (0.15 M NaCl, 5 mM sodium phosphate buffer, pH 7.4). Frozen tissue pieces were drenched in 10 mM sodium phosphate buffer (pH 6.5) containing 0.1 mM phenylmethylsulphonylfluorid (PMSF). The surface and sub-mucosa of normal tissue was removed by scraping the tissue with a glass microscope slide. Tumor tissue was homogenized without prior scraping. The material was immersed in liquid nitrogen and pulverized in a Retch tissue pulverizer (Retch GmBH& Co., Haan, Germany), then placed into five volumes of extraction buffer (6 M guanidinium chloride, 5mM ethylenediaminetetraacetic acid (EDTA), 10 mM sodium phosphate buffer, pH 6.5) containing 0.1 mM PMSF and dispersed using a Dounce homogenizer (four strokes with a loose pestle) and stirred slowly at 4°C overnight. The insoluble material was removed by centrifugation at 23,000 × g for 50 min at 4°C (Beckman JA-30 rotor). Supernatants were dialyzed against ten volumes of extraction buffer for 24 h and then filled up to 26 mL with extraction buffer. Cesium chloride was added to the samples to give 1.39 g/mL starting density by gentle stirring. The samples were centrifuged at 40,000 × g for 90 h at 15°C. The fractions were collected from the bottom of the tubes with a fraction collector equipped with a drop counter. Density measurements were performed using a Carlsberg pipette as a pycnometer. UV light absorbance at 280 nm demonstrated that DNA and low density proteins were in other fractions than the high density mucins. The glycan structures on these mucins are also available in the supplementary table S1.

### Detection of carbohydrates as a measure of mucin concentration

Density gradient fractions of purified mucin samples were analyzed for carbohydrates as periodate-oxidizable structures in a microtiter-based assay. Polysorp 96-well plates (ThermoFisher Scientific, 475094) were coated with mucin sample and kept overnight at 4°C. After washing three times with a washing solution (5mM Tris-HCl, 0.15 M NaCl, 0.005% Tween 20, 0.02% NaN_3_, pH 7.75), the carbohydrates were oxidized by treatment with 25 mM sodium metaperiodate in 0.1 M sodium acetate buffer, pH 5.5 for 20 min at room temperature (RT). The plates were washed again and the wells were blocked with DELFIA (dissociation-enhanced lanthanide fluoroimmunoassay) blocking solution (50 mMTris-HCl, 0.15 M NaCl, 90 µM CaCl_2_, 4 µM EDTA, 0.02% NaN_3_, 6% sorbitol, 0.1% bovine serum albumin (BSA), pH 7.75) for 1 h. After further washing, the samples were incubated for 1 h with 2.5 µM biotin hydrazide in 0.1 M sodium acetate buffer, pH 5.5, followed by washing. Europium-labeled streptavidin was diluted 1/400 in assay buffer (50 mMTris-HCl, 0.15 M NaCl, 20 µM diethylenetriaminepentaacetic acid, 0.01% Tween 20, 0.02% NaN_3_, 1.5% BSA, pH 7.75) and was added to the wells. After 1 h incubation, the plates were washed six times and then incubated with enhancement solution (0.05 M NaOH, 0.1 M phthalate, 0.1% Triton X-100, 50 µM tri-n-octylphosphine oxide, 15 µM β-2-naphthoyltrifluoroacetone) for 5 min on a shaker. Fluorescence was measured (λex=340 nm and λem=615 nm) using a Wallac 1420 VICTOR2 plate reader with the Europium label protocol (PerkinElmer, Waltham, MA, USA). Gradient fractions containing mucins were pooled together. Mucin concentration in pooled samples was determined by the same method including a standard curve of a fusion protein of MUC1, 16TR and IgG2a Fc starting at a concentration of 20 µg/mL using seven 1/2 serial dilutions. Pooled samples were dialysed (cellulose membrane, molecular weight cut-off 14 kDa Sigma-Aldrich, St. Louise, MO, USA) against sterile PBS prior to use in bacterial cultures.

### Enzyme-linked immunosorbent assays for glycan and mucin determination

Mucin samples were analyzed for the relative content of Le^b^, SLe^a^, SLe^x^, α1,4-GlcNAc, MUC5AC, MUC6, MUC2 and MUC5B. The mucin samples were diluted in 4 M GuHCl to 6 µg/mL for the glycosylation analysis or to 3 µg/mL for the mucin analysis and coated in 96-well Polysorp plates overnight at 4°C. The samples analyzed with LUM6-3, LUM2-3 and LUM5B-2 were reduced with 2 mM 1,4-dithiothreitol in 6 M GuHCl, 5 mM EDTA, 0.1 M Tris-HCl buffer, pH 8.0, at 37°C for 1 h and alkylated in 5 mM iodoacetamide at RT for 1 h in the dark to expose the epitopes. All plates were washed three times with PBS 0.05% Tween (washing buffer) and the wells were blocked for 1 h with Blocking Reagent for ELISA (Roche, 11112589001) containing 0.05% Tween (blocking buffer) at RT. After discarding the blocking buffer, the plates were incubated for 1 h with Seraclone anti-Le^b^ (Biotest, clone LE2), anti-sialyl-Le^a^ (NeoMarkers, clone CA19-9), anti-sialyl-Le^x^ (AM3, courtesy of Dr. C. Hanski, University Medical Center Charité, Berlin, Germany) diluted to 1 mg/mL, 1/200, 1/1000 and 1/20, respectively, and anti-α1,4-GlcNAc (Kanto Chemical Co., Inc., HIK1083) diluted 1/50, anti-MUC5AC (Sigma-Aldrich, 45M1) diluted 1/8000, anti-MUC6 (LUM6-3) [40], anti-MUC2 (LUM2-3) [41], and anti-MUC5B (LUM5B-2) [42] diluted 1/2000 in blocking buffer. The plates were washed three times and then incubated for 1 h with 0.8 µg/mL horseradish peroxidase (HRP) conjugated anti-mouse IgM, anti-mouse IgG or anti-rabbit IgG diluted in blocking buffer. After washing, 100 µL tetramethylbenzidine (TMB) substrate (Sigma-Aldrich, T0440) was added to the wells and the plates were monitored for color development. The reaction was stopped with an equivalent amount of 0.5 M H_2_SO_4_ and the absorbance at 450 nm was measured. The mucin density, profile, and glycan structures are summarized in Table 2.

### Mucin glycan identification by mass spectrometry

*O*-glycans were released and analyzed as described previously [26]. In brief, *O*-glycans were released by reductive β-elimination. Released glycans were analyzed by LC-MS using a 10 cm × 250 μm I.D. column, prepared in-house, containing 5 μm porous graphitized carbon particles (Thermo Scientific, Waltham, MA, USA). Glycans were eluted using a linear gradient from 0–40% acetonitrile in 10 mM NH_4_HCO_3_ over 40 min at a flow rate of 10 μL/min. The eluted glycans were detected using a LTQ ion trap mass spectrometer (Thermo Scientific) in negative ion mode with an electro spray voltage of 3.5 kV, a capillary voltage of −33.0 V, and a capillary temperature of 300°C. Air was used as sheath gas and mass ranges were defined dependent on the specific structure to be analyzed. The data were processed using the X calibur software (version 2.0.7, Thermo Scientific). Glycans were identified from their MS/MS spectra by manual annotation. Diagnostic fragmentation ions for *O*-glycans were investigated as described [43]. The annotated structures and associated MIRAGE (the Minimum Information Required for a Glycomics Experiment) are available at the UniCarb-DR database (http://unicarb-dr.biomedicine.gu.se/references/348).

### Helicobacter pylori clinical strains

Twenty *H. pylori* strains were isolated from biopsies obtained from Portuguese children aged 1 to 15 years. Nine *H. pylori* strains were collected from patients with abdominal pain and gastritis (non-ulcer dyspepsia, NUD), and the remaining 11 strains were isolated from children with gastric or duodenal ulcers (PUD). Patient data and *H. pylori* strains included in this study are presented in Table 1.

### Genotyping of H. pylori virulence genes

All of the NUD and PUD *H. pylori* pediatric strains were genotyped for the presence/status of *babA, babB, babC, cagA, vacAs*allele, *hopQ* allele, *oipA, sabA, hopZ*, and *homB* outer membrane protein encoding genes (Table 1) by whole genome sequencing (WGS), considering the previously published 12 *H. pylori* genomes [44,45], and eight new genomes, that were sequenced on a MiSeq Illumina platform, as previously described [45]. Briefly, high-quality genomic DNA samples from pure bacterial cultures were used to prepare Nextera XT Illumina libraries that were sequenced on an Illumina MiSeq platform (Illumina Inc., San Diego, CA) using the v.2 (300 cycles, 2×150 nt reads or 500 cycles, 2×250 nt reads) kit, according to the manufacturer’s instructions. FastQC v0.11.3 (http://www.bioinformatics.babraham.ac.uk/projects/fastqc/) and FASTX v0.0.13 (http://hannonlab.cshl.edu/fastx_toolkit/) software tools were applied to evaluate and improve the quality of the raw read sequence data, respectively. Draft genomes were *de novo* assembled using SPAdes v3.7.1. The Rapid Annotation using Subsystem Technology (RAST) server was used for annotation of the whole-genome in order to identify presence/status of the previously described virulence genes [46]. Raw sequence reads of the genomes of *H. pylori* strains have been deposited in the Sequence Read Archive (SRA) (accession numbers are provided in Table 1).

### Construction of H. pylori babA and homB knockout mutant strains

The *homB* and *babA* knockout mutant strains were constructed by insertion of a kanamycin cassette by natural transformation and double homologous recombination, as previously described [4]. Seven *H. pylori* clinical strains were used for *homB* knockouts, two NUD strains (565/99, 36/00) and five DU strains (417/02, 1089/03, 771/99, 559/02 and 1776/05), while for *babA* knockout three DU strains were used (559/02, 1776/05, 441/02).The correct and unique insertion of the antibiotic cassette was confirmed by NGS for two *babA* mutant strains (1776/05 and 441/02), as described above, and by Southern-blot for the remaining mutants, as previously described [4].

### Bacterial culture conditions

*H. pylori* strains were cultured on *Brucella* medium base (Oxoid, CM0169B) supplemented with 10% bovine blood, 1% Vitox supplement (Oxoid,SR0090H), 4 mg/L Amphotericin B, 10 mg/L Vancomycin and 5 mg/L Trimethoprim in a microaerophilic environment (5% O2 and 15% CO2) at 37°C.

### Binding of H. pylori to mucins and glycoconjugates

Human and monkey gastric mucin samples, DNA, and carbohydrate structures Le^b^, SLe^x^, and Lactose-N-tetraose (LNT) conjugated to human serum albumin (IsoSep AB, 61/08, 61/66, and 60/97 respectively), were diluted to 4 µg/mL and coated overnight at 4°C onto Polysorp96-well plates. The plates were washed three times with PBS containing 0.05% Tween and blocked for 1 h with 1% blocking reagent for ELISA (Roche, 11112589001). After discarding the blocking buffer, bacteria with an OD_600_ of 0.1 were diluted 1/10 in a solution containing 1% blocking reagent for ELISA (1% protein w/v in 50mM Tris-HCl and 150 mMNaCl), 0.05% Tween, and 10 mM citric acid, set to pH 2.0, 4.0 and 7.0. Changing the pH from 7.0 to 2.0 changes the osmolarity of the solution by aproximately 0.25%. A 100 µL of bacterial suspension were added to the plates, and incubated at 37°C for 2 h with shaking. The plates were washed three times with PBS, followed by incubation with a rabbit anti-*H. pylori* serum (kindly provided by Thomas Bóren, Umeå University) diluted 1/1000 for 1 h at RT. Three more washes with PBS containing 0.05% Tween were performed before and after wells were incubated with a horse radish peroxidase (HRP) conjugated donkey anti-rabbit IgG (Jackson ImmunoResearch, West Grove, PA, USA) diluted 1/10000 for 1 h. Subsequently, 100µL of tetramethylbenzidine substrate (Sigma-Aldrich, T0440) was added per well, and the reaction was stopped with an equivalent volume of 0.5 M H_2_SO_4_. Absorbance at 450 nm was measured in a Wallac 1420 VICTOR2 plate reader.

### H. pylori proliferation in co-culture with mucins and le^b^

*H. pylori* were harvested from agar plates into PBS, centrifuged at 2500* g* for 5 min, and resuspended in *Brucella* broth containing 10 mM urea and 20% FBS to an OD_600_ of 0.1. The bacterial suspension and dialyzed human mucins (3IS: Le^b+^/ɑ1,4-GlcNAc^−^, and 4ID: Le^b-^/ɑ1,4-GlcNAc^+^) or Le^b^ (IsoSep AB) diluted in PBS (100 µg/mL), were added to a volume of 100 µL per well in a 96-well plate. Proliferation control wells contained PBS instead of mucins in addition to the culture medium. To ensure residual CsCl and GuHCl had no adverse effects on *H. pylori* growth, a control consisting of CsCl dissolved in 4 M GuHCl was dialyzed in parallel to the mucins, and had similar results on *H. pylori* growth compared to the PBS control. The plate was covered and incubated at 37°C in a microaerophilic environment for 4 h. One hundred microliters of the RealTime-Glo cell viability assay (Promega, G9712) diluted 1/500 in *Brucella* broth containing 10 mM urea and 20% FBS were added per well, resulting in a final OD_600_ of 0.05 and final volume of 200 µL. The assay was performed at pH 5 and 7. The microtiter plate was covered with a sterile gas permeable sealing membrane (Diversified Biotech) and incubated at 37°C inside a plate reader (CLARIOstar, BGM labtech GmbH, Ortenberg, Germany) connected to an atmospheric unit set to 5% O_2_ and 10% CO_2_ for 24 h. Luminescence readings were measured every 2 h for 24 h.

### Statistics

Statistical analysis was performed using GraphPad Prism version 6 software (La Jolla, CA, USA). Results are expressed as the mean ± standard errors of the means (SEM) for normally distributed data, and median with interquartile range (IQR) for data that did not follow a normal distribution (determined using the D’Agostino-Pearson omnibus test). Data were analyzed using the Student’s *t*, Mann-Whitney U, Kruskal-Wallis or One-way ANOVA tests wherever applicable, and *p* values ≤ 0.05 were considered as statistically significant.

## Abbreviations

*NUD*: Non-ulcer dyspepsia
*PUD*: Peptic ulcer disease
*Le^b^*: Lewis b
*SLe^x^*: Sialyl-Lewis x
*SLe^a^*: Sialyl-Lewis a
*LNT*: Lacto-N-tetraose
*LacdiNAc*: GalNAcβ1-4GlcNAc
*H. pylori*: *Helicobacter pylori*
*H. suis*: *Helicobacter suis*
*MALT*: Mucosa-associated lymphoid tissue
*DU*: Duodenal ulcer
*GU*: Gastric ulcer
*SabA*: Sialic acid binding adhesin
*BabA*: Blood group antigen binding adhesin
*cagA*: Cytotoxin-associated gene A
*vacA*: Vacuolating cytotoxin gene A
*hopQ*: *Helicobacter pylori* outer membrane protein Q
*oipA*: Outer inflammatory protein A
*hopZ*: *Helicobacter pylori* outer membrane protein Z
*homB*: *Helicobacter pylori* outer membrane adhesion molecule B
*WGS*: Whole genome shotgun
*OMPs*: Outer membrane proteins
*IS*: Insoluble surface
*ID*: Insoluble deep
*EDTA*: Ethylenediaminetetraacetic acid
*PMSF*: Phenylmethylsulfonyl fluoride
*DELFIA*: Dissociation-enhanced lanthanide fluoroimmunoassay
*BSA*: Bovine serum albumin
*ELISA*: Enzyme-linked immunosorbent assay
*TMB*: 3,3′,5,5′-Tetramethylbenzidine
RAST: Rapid Annotation using Subsystem Technology
*SRA*: Sequence Read Archive
*LC-MS*: Liquid chromatography–mass spectrometry
*HRP*: Horseradish peroxidase
*CsCl*: Cesium chloride
*GuHCl*: Guanidinium hydrochloride

## Supplementary material

Supplementary material data can be accesssed here.

## References

[1] Lai LH, Sung JJ. Helicobacter pylori and benign upper digestive disease. Best Pract Res Clin Gastroenterol. 2007;21(2):261–279. PubMed PMID: 17382276. 17382276 10.1016/j.bpg.2006.10.002

[2] Atherton JC The pathogenesis of Helicobacter pylori-induced gastro-duodenal diseases. Annu Rev Pathol. 2006;1:63–96. PubMed PMID: 18039108. 18039108 10.1146/annurev.pathol.1.110304.100125

[3] Konturek PC, Konturek SJ, Brzozowski T Helicobacter pylori infection in gastric cancerogenesis. J Physiol Pharmacol. 2009 9;60(3):3–21. PubMed PMID: 19826177. 19826177

[4] Oleastro M, Cordeiro R, Ferrand J, et al Evaluation of the clinical significance of homB, a novel candidate marker of Helicobacter pylori strains associated with peptic ulcer disease. J Infect Dis. 2008 11 1;198(9):1379–1387. PubMed PMID: 18811585. 18811585 10.1086/592166

[5] Gerhard M, Lehn N, Neumayer N, et al Clinical relevance of the Helicobacter pylori gene for blood-group antigen-binding adhesin. Proc Natl Acad Sci U S A. 1999;96(22):12778–12783. 10535999 10.1073/pnas.96.22.12778 PMC23096

[6] Oleastro M, Cordeiro R, Yamaoka Y, et al Disease association with two Helicobacter pylori duplicate outer membrane protein genes, homB and homA. Gut Pathog. 2009 6 22;1(1):12 PubMed PMID: 19545429; PubMed Central PMCID: PMCPMC2706848. 19545429 10.1186/1757-4749-1-12 PMC2706848

[7] Buisine MP, Devisme L, Degand P, et al Developmental mucin gene expression in the gastroduodenal tract and accessory digestive glands. II. Duodenum and liver, gallbladder, and pancreas. J Histochem Cytochem. 2000 12;48(12):1667–1676. PubMed PMID: 11101635. 11101635 10.1177/002215540004801210

[8] Linden SK, Sutton P, Karlsson NG, et al Mucins in the mucosal barrier to infection. Mucosal Immunol. 2008 5;1 3:183–197. PubMed PMID: 19079178; eng. 19079178 10.1038/mi.2008.5 PMC7100821

[9] Aspholm-Hurtig M, Dailide G, Lahmann M, et al Functional adaptation of BabA, the H. pylori ABO blood group antigen binding adhesin. Science. 2004 7 23;305(5683):519–522. PubMed PMID: 15273394. 15273394 10.1126/science.1098801

[10] Lindén S, Nordman H, Hedenbro J, et al Strain- and blood group-dependent binding of Helicobacter pylori to human gastric MUC5AC glycoforms. Gastroenterology. 2002;123(6):1923–1930. 12454849 10.1053/gast.2002.37076

[11] Linden SK, Wickstrom C, Lindell G, et al Four modes of adhesion are used during Helicobacter pylori binding to human mucins in the oral and gastric niches. Helicobacter. 2008 4;13(2):81–93. PubMed PMID: 18321298; eng. 18321298 10.1111/j.1523-5378.2008.00587.x

[12] Rossez Y, Gosset P, Boneca IG, et al The LacdiNAc-Specific Adhesin LabA Mediates Adhesion of Helicobacter pylori to Human Gastric Mucosa. J Infect Dis. 2014 10 15;210(8):1286–1295. PubMed PMID: 24755437. 24755437 10.1093/infdis/jiu239

[13] Padra M, Adamczyk B, Benktander J, et al Helicobacter suis binding to carbohydrates on human and porcine gastric mucins and glycolipids occurs via two modes. Virulence. 2018;1–48. DOI:10.1080/21505594.2018.1460979. 29638186 PMC5955484

[14] Ho SB, Shekels LL, Toribara NW, et al Mucin gene expression in normal, preneoplastic, and neoplastic human gastric epithelium. Cancer Res. 1995 6 15;55(12):2681–2690. PubMed PMID: 7780985. 7780985

[15] Byrd JC, Yan P, Sternberg L, et al Aberrant expression of gland-type gastric mucin in the surface epithelium of Helicobacter pylori-infected patients. Gastroenterology. 1997;113(2):455–464. 9247464 10.1053/gast.1997.v113.pm9247464

[16] Tsai CJ, Herrera-Goepfert R, Tibshirani RJ, et al Changes of gene expression in gastric preneoplasia following Helicobacter pylori eradication therapy. Cancer Epidemiol Biomarkers Prev. 2006 2;15(2):272–280. PubMed PMID: 16492915. 16492915 10.1158/1055-9965.EPI-05-0632

[17] Sakamoto S, Watanabe T, Tokumaru T, et al Expression of Lewisa, Lewisb, Lewisx, Lewisy, siayl-Lewisa, and sialyl-Lewisx blood group antigens in human gastric carcinoma and in normal gastric tissue. Cancer Res. 1989 2 1;49(3):745–752. PubMed PMID: 2910493. 2910493

[18] Cooke CL, An HJ, Kim J, et al Modification of gastric mucin oligosaccharide expression in rhesus macaques after infection with Helicobacter pylori. Gastroenterology. 2009 9;137(3):1061-71, 1071 e1-8 PubMed PMID: 19375420. 10.1053/j.gastro.2009.04.014 19375420

[19] Linden S, Mahdavi J, Semino-Mora C, et al Role of ABO secretor status in mucosal innate immunity and H. pylori infection. PLoS Pathog. 2008 1;4 1:e2 PubMed PMID: 18179282; PubMed Central PMCID: PMC2174967. eng. 18179282 10.1371/journal.ppat.0040002 PMC2174967

[20] Liu H, Fero JB, Mendez M, et al Analysis of a single Helicobacter pylori strain over a 10-year period in a primate model. Int J Med Microbiol. 2015 5;305(3):392–403. PubMed PMID: 25804332; PubMed Central PMCID: PMCPMC4376324. 25804332 10.1016/j.ijmm.2015.03.002 PMC4376324

[21] Linden SK, Sheng YH, Every AL, et al MUC1 limits Helicobacter pylori infection both by steric hindrance and by acting as a releasable decoy. PLoS Pathog. 2009 10;5(10):e1000617 PubMed PMID: 19816567; PubMed Central PMCID: PMC2752161. eng. 19816567 10.1371/journal.ppat.1000617 PMC2752161

[22] Benktander J, Angstrom J, Breimer ME, et al Redefinition of the carbohydrate binding specificity of Helicobacter pylori BabA adhesin. J Biol Chem. 2012 9 14;287(38):31712–31724. PubMed PMID: 22822069; PubMed Central PMCID: PMC3442506. 22822069 10.1074/jbc.M112.387654 PMC3442506

[23] Linden S, Semino-Mora C, Liu H, et al Role of mucin Lewis status in resistance to Helicobacter pylori infection in pediatric patients. Helicobacter. 2010 8;15(4):251–258. PubMed PMID: 20633185; eng. 20633185 10.1111/j.1523-5378.2010.00765.x PMC3209514

[24] Navabi N, Johansson ME, Raghavan S, et al Helicobacter pylori Infection Impairs the Mucin Production Rate and Turnover in the Murine Gastric Mucosa. Infect Immun. 2013 3;81(3):829–837. PubMed PMID: 23275091; PubMed Central PMCID: PMC3584886. 23275091 10.1128/IAI.01000-12 PMC3584886

[25] Vitoriano I, Saraiva-Pava KD, Rocha-Goncalves A, et al Ulcerogenic Helicobacter pylori strains isolated from children: a contribution to get insight into the virulence of the bacteria. PLoS One. 2011;6(10):e26265 PubMed PMID: 22039453; PubMed Central PMCID: PMCPMC3198394. 22039453 10.1371/journal.pone.0026265 PMC3198394

[26] JinC, KennyDT, SkoogEC, et al Structural Diversity of Human Gastric Mucin Glycans. Mol Cell Proteomics. 2017 5;16(5):743–758. PubMed PMID: 28461410; PubMed Central PMCID: PMCPMC5417818.2846141010.1074/mcp.M117.067983

[27] Linden S, Mahdavi J, Hedenbro J, et al Effects of pH on Helicobacter pylori binding to human gastric mucins: identification of binding to non-MUC5AC mucins. Biochem J. 2004 12 1;384(Pt 2):263–270. PubMed PMID: 15260802. 15260802 10.1042/BJ20040402 PMC1134109

[28] Skoog EC, Padra M, Aberg A, et al BabA dependent binding of Helicobacter pylori to human gastric mucins cause aggregation that inhibits proliferation and is regulated via ArsS. Sci Rep. 2017 1;20(7):40656 PubMed PMID: 28106125; PubMed Central PMCID: PMCPMC5247751. 28106125 10.1038/srep40656 PMC5247751

[29] Skoog EC, Sjoling A, Navabi N, et al Human Gastric Mucins Differently Regulate Helicobacter pylori Proliferation, Gene Expression and Interactions with Host Cells. PLoS One. 2012;7(5):e36378 10.1371/journal.pone.0036378 PONE-D-11-18652 PubMed PMID: 22563496; PubMed Central PMCID: PMC3341350. eng. 22563496 10.1371/journal.pone.0036378 PMC3341350

[30] Kawakubo M, Ito Y, Okimura Y, et al Natural antibiotic function of a human gastric mucin against Helicobacter pylori infection. Science. 2004 8 13;305(5686):1003–1006. PubMed PMID: 15310903. 15310903 10.1126/science.1099250

[31] Kivi M, Tindberg Y, Sorberg M, et al Concordance of Helicobacter pylori strains within families. J Clin Microbiol. 2003 12;41(12):5604–5608. PubMed PMID: 14662948; PubMed Central PMCID: PMCPMC309035. 14662948 10.1128/JCM.41.12.5604-5608.2003 PMC309035

[32] Oleastro M, Monteiro L, Lehours P, et al Identification of markers for Helicobacter pylori strains isolated from children with peptic ulcer disease by suppressive subtractive hybridization. Infect Immun. 2006 7;74(7):4064–4074. PubMed PMID: 16790780; PubMed Central PMCID: PMCPMC1489719. 16790780 10.1128/IAI.00123-06 PMC1489719

[33] Oleastro M, Santos A, Cordeiro R, et al Clinical relevance and diversity of two homologous genes encoding glycosyltransferases in Helicobacter pylori. J Clin Microbiol. 2010 8;48(8):2885–2891. PubMed PMID: 20554820; PubMed Central PMCID: PMCPMC2916562. 20554820 10.1128/JCM.00401-10 PMC2916562

[34] Park JS, Yeom JS, Seo JH, et al Immunohistochemical Expressions of MUC2, MUC5AC, and MUC6 in Normal, Helicobacter pylori Infected and Metaplastic Gastric Mucosa of Children and Adolescents. Helicobacter. 2015 8;20(4):260–268. PubMed PMID: 25704078. 25704078 10.1111/hel.12198

[35] Bugaytsova JA, Bjornham O, Chernov YA, et al Helicobacter pylori Adapts to Chronic Infection and Gastric Disease via pH-Responsive BabA-Mediated Adherence. Cell Host Microbe. 2017 3 8;21(3):376–389. PubMed PMID: 28279347; PubMed Central PMCID: PMCPMC5392239. 28279347 10.1016/j.chom.2017.02.013 PMC5392239

[36] Hunt RH, Camilleri M, Crowe SE, et al The stomach in health and disease. Gut. 2015 10;64(10):1650–1668. PubMed PMID: 26342014; PubMed Central PMCID: PMCPMC4835810. 26342014 10.1136/gutjnl-2014-307595 PMC4835810

[37] McAuley JL, Linden SK, Png CW, et al MUC1 cell surface mucin is a critical element of the mucosal barrier to infection. J Clin Invest. 2007 8;117(8):2313–2324. PubMed PMID: 17641781; PubMed Central PMCID: PMC1913485. eng. 17641781 10.1172/JCI26705 PMC1913485

[38] McGuckin MA, Every A, Skene C, et al Muc1 mucin limits both Helicobacter pylori colonization of the murine gastric mucosa and the associated gastritis. Gastroenterology. 2007;133(4):1210–1218. 17919495 10.1053/j.gastro.2007.07.003

[39] Linden S, Boren T, Dubois A, et al Rhesus monkey gastric mucins: oligomeric structure, glycoforms and Helicobacter pylori binding. Biochem J. 2004 5 1;379(Pt 3):765–775. 10.1042/BJ20031557 BJ20031557 PubMed PMID: 14736333; PubMed Central PMCID: PMC1224112. eng. 14736333 10.1042/BJ20031557 PMC1224112

[40] Nordman H, Davies JR, Lindell G, et al Gastric MUC5AC and MUC6 are large oligomeric mucins that differ in size, glycosylation and tissue distribution. Biochem J. 2002 5 15;364(Pt 1):191–200. PubMed PMID: 11988092. 11988092 10.1042/bj3640191 PMC1222561

[41] Herrmann A, Davies JR, Lindell G, et al Studies on the “insoluble” glycoprotein complex from human colon. Identification of reduction-insensitive MUC2 oligomers and C-terminal cleavage. J Biol Chem. 1999;274(22):15828–15836. 10336486 10.1074/jbc.274.22.15828

[42] Wickstrom C, Davies JR, Eriksen GV, et al MUC5B is a major gel-forming, oligomeric mucin from human salivary gland, respiratory tract and endocervix: identification of glycoforms and C-terminal cleavage. Biochem J. 1998 9 15;334(Pt 3):685–693. PubMed PMID: 9729478; PubMed Central PMCID: PMCPMC1219739. 9729478 10.1042/bj3340685 PMC1219739

[43] Everest-Dass AV, Abrahams JL, Kolarich D, et al Structural feature ions for distinguishing N- and O-linked glycan isomers by LC-ESI-IT MS/MS. J Am Soc Mass Spectrom. 2013 6;24(6):895–906. PubMed PMID: 23605685. 23605685 10.1007/s13361-013-0610-4

[44] Silva B, Nunes A, Vale FF, et al The expression of Helicobacter pylori tfs plasticity zone cluster is regulated by pH and adherence, and its composition is associated with differential gastric IL-8 secretion. Helicobacter. 2017 8;22(4):e12390 PubMed PMID: 28436598. 10.1111/hel.12390 28436598

[45] Nunes A, Rocha R, Vale FF, et al Genome Sequencing of 10 Helicobacter pylori Pediatric Strains from Patients with Nonulcer Dyspepsia and Peptic Ulcer Disease. Genome Announc. 2015 2 05;3(1). e01488–14. PubMed PMID: 25657274; PubMed Central PMCID: PMCPMC4319599. 25657274 10.1128/genomeA.01488-14 PMC4319599

[46] Aziz RK, Bartels D, Best AA, et al The RAST Server: rapid annotations using subsystems technology. BMC Genomics. 2008 2;8(9):75 PubMed PMID: 18261238; PubMed Central PMCID: PMCPMC2265698. 18261238 10.1186/1471-2164-9-75 PMC2265698

